# Investigating Mental Health of US College Students During the COVID-19 Pandemic: Cross-Sectional Survey Study

**DOI:** 10.2196/22817

**Published:** 2020-09-17

**Authors:** Xiaomei Wang, Sudeep Hegde, Changwon Son, Bruce Keller, Alec Smith, Farzan Sasangohar

**Affiliations:** 1 Industrial and Systems Engineering Texas A&M University College Station, TX United States; 2 Center for Outcomes Research Houston Methodist Hospital Houston, TX United States

**Keywords:** mental health, online survey, COVID-19, coronavirus, college student, student, stress, depression, university

## Abstract

**Background:**

Evidence suggests that the COVID-19 pandemic has generally increased levels of stress and depression among the public. However, the impact on college students in the United States has not been well-documented.

**Objective:**

This paper surveys the mental health status and severity of depression and anxiety of college students in a large university system in the United States during the COVID-19 pandemic.

**Methods:**

An online survey was conducted among undergraduate and graduate students recruited from Texas A&M University via email. The survey consisted of two standardized scales—the Patient Health Questionnaire-9 and the General Anxiety Disorder-7—for depression and anxiety, and additional multiple-choice and open-ended questions regarding stressors and coping mechanisms specific to COVID-19.

**Results:**

Among the 2031 participants, 48.14% (n=960) showed a moderate-to-severe level of depression, 38.48% (n=775) showed a moderate-to-severe level of anxiety, and 18.04% (n=366) had suicidal thoughts. A majority of participants (n=1443, 71.26%) indicated that their stress/anxiety levels had increased during the pandemic. Less than half of the participants (n=882, 43.25%) indicated that they were able to cope adequately with the stress related to the current situation.

**Conclusions:**

The proportion of respondents showing depression, anxiety, and/or suicidal thoughts is alarming. Respondents reported academic-, health-, and lifestyle-related concerns caused by the pandemic. Given the unexpected length and severity of the outbreak, these concerns need to be further understood and addressed.

## Introduction

The United States has seen a surge in the number of COVID-19 cases since March 2020, with initial peaks in April 2020 [1]. Recent assessments of mental health in the general populace of China and Iran, countries that had major outbreaks, show increased levels of stress due to the pandemic [2,3]. A key concern during the pandemic relates to the mental health of vulnerable populations, including college students. The 2019 Annual Report of the Center for Collegiate Mental Health [4] reported that anxiety continues to be the most common problem (62.7% of 82,685 respondents) among students who completed the Counseling Center Assessment of Psychological Symptoms. Consistent with the national trend, Texas A&M University has seen an increase in the number of students seeking services for anxiety disorders over the last few years. Given the vulnerability of this population during the pandemic, there is a critical need to assess the mental health of college students in order to address concerns in a timely manner [5-8].

Recent assessments of college student mental health in China have shown an increased level of anxiety and depression in the wake of the pandemic [6,9]. These studies used standardized scales for depression and anxiety, such as the depression and anxiety stress scale, the Patient Health Questionnaire-9 (PHQ-9), and the Generalized Anxiety Disorder-7 (GAD-7) questionnaire. General panic related to the outbreak and risk of exposure were found to be contributors to increased level of depression. Similarly, Cao et al [9] assessed anxiety using the GAD-7 scale and found that risk of infection, including for family members, was a major contributor to college students’ increased anxiety. Both studies also identified important protective factors such as income stability and the availability of information related to preventative measures. However, these studies do not include an assessment of strategies used by students themselves for coping with and managing their stress. Additionally, these studies have focused on the student population in China. Given the differences in cultural, geographic, economic, and other factors, an assessment of college students’ mental health in the United States is needed.

The aim of this study was to conduct a survey-based assessment of mental health among college students at Texas A&M University, a large university in the United States, during the COVID-19 pandemic. We sought to identify severity levels of depression and anxiety symptoms (primary outcome), as well as stressors related to the pandemic, coping mechanisms used, and barriers experienced by students in handling pandemic-related stress.

## Methods

### Recruitment

An online cross-sectional survey was designed and conducted during the initial peak of the COVID-19 pandemic in the late Spring 2020 semester at Texas A&M. The research received approval from the university’s institutional review board. Guidelines provided by Kelley et al [10] were used for designing, conducting, and reporting this survey research.

Participants were recruited from the student population. This university closed all campuses on March 23, 2020, and held all classes virtually in response to the COVID-19 pandemic. In addition, the State of Texas issued a stay-at-home order on April 2, 2020. The survey was published using the online survey platform Qualtrics on May 4, 2020, and data collection remained open until no additional completion was reported for two days (May 19, 2020). During this period, the survey was announced to the entire population of over 60,000 students at the Texas A&M College Station Campus, through email.

### Survey Design

The survey was designed in a semistructured format comprising multiple-choice questions and free-text fields for elaboration. The survey consisted of the following five sections:

#### Demographics

This section included questions related to participants’ age, gender, college classification (ie, undergraduate [freshman, sophomore, junior, senior] or graduate [master’s, doctorate]), and program of study.

#### Patient Health Questionnaire

The PHQ-9 is a validated and widely used measure of depression severity in primary and mental health care, consisting of 9 items based on depression symptoms. Respondents report the frequency of symptoms experienced within the last 2 weeks. The categories of severity range are minimal (0-4), mild (5-9), moderate (10-14), moderately severe (15-19), and severe (20-27) [11].

#### Generalized Anxiety Disorder Screener

The GAD-7 is a validated questionnaire used in most mental health care settings as a screening tool for major anxiety disorders such as generalized anxiety disorder or panic disorder [12], consisting of 7 items based on GAD symptoms. Respondents rate the frequency of experiencing these symptoms within the last 2 weeks. The categories of severity range are minimal (0-4), mild (5-9), moderate (10-14), and severe (15-21).

#### Questions Related to COVID-19–Related Stress

This section aimed at identifying various stressors resulting from the pandemic and comprised the following items:

- Did your overall stress increase/decrease or remain the same during the ongoing pandemic? (response: increase/decrease/remain the same)
  - If the participant chose increase/decrease, a follow-up open-answer question was asked: Can you describe the main reason for such increase/decrease of stress and anxiety?
- Do you think other students are experiencing stress/anxiety because of the pandemic? (response: yes/no)
- In the past month, what level of fear, worry, and/or changes have you experienced related to any of the following academic/health/lifestyle-related concerns? (response: none/mild/moderate/severe)
- Did you have any other academic/health/lifestyle-related concerns? (response: yes/no)
  - If participant chose “yes,” a follow-up open-answer question was asked: Please specify any other academic-related concerns you have.

#### Coping Mechanisms and Barriers

This section consisted of multiple-choice questions and open-ended follow-up questions related to ways in which the students coped with stress during the pandemic. Follow-up questions were included to identify specific resources and technological apps that students may have been using as they coped:

- Do you feel you are able to cope adequately with the stress related to the current situation? (response: yes/no/maybe)
- What coping methods/tools/techniques have you used to mitigate your elevated stress/anxiety? (response: none; university services; health services outside the university; support from community, family and friends; technologies; other)
  - If participant chose “university services,” a follow-up question was asked: What university services did you use because of the pandemic? (response: student health services; counseling and psychological services; other)
- Where are you sourcing your information [about the pandemic] from? (response: university emails; your medical provider; newspaper and periodicals; medical websites; posts on social media; other)
- Have you been using any mobile apps or features on existing apps for managing stress, anxiety, or depression related to the ongoing COVID-19 pandemic? (response: yes/no)
  - If participant chose “yes,” a follow-up open-answer question was asked: Please specify what apps/features you have been using.
- In your opinion, what are the barriers to mental health care? (response: none; lack of information about resources available; financial concerns; limited access to the services; social stigma; other)

### Data Analysis

For PHQ-9 and GAD-7, mean scores were calculated for different gender and classification groups. The percentages of participants who fall in each severity category were computed. Inspection of the data and residual plots for mean PHQ-9 and GAD-7 scores did not indicate any violation of assumptions of normality, independence, and homogeneity of variance. Therefore, a two-way analysis of variance (ANOVA) was conducted to identify significant main effects and interactions between gender and classification groups. For the questions regarding concerns about COVID-19, the percentage of participants who chose each severity level was computed. For the multiple-choice questions regarding coping mechanisms and barriers, the percentage of participants who chose each item was computed. Quantitative analyses were performed using Microsoft Excel (Microsoft Corp) and R 4.0.2 (The R Foundation).

Open-ended questions were coded using thematic analysis [13]. Initial codes were created based on a previous coding scheme used for an interview study [14]. Initial coding consisted of placing all responses to the questions into the initial codes; however, responses that did not fit in the initial codes were placed in new codes generated inductively. Focused coding, following initial coding, consisted of recategorizing codes and creating additional codes as needed. For the final phase of thematic coding, common themes were identified among the codes and numbers of appearance were counted. The analysis of the open-ended questions was split among four coders: one coder analyzed the questions related to increased or decreased stress (BK); one coder analyzed the questions related to academic concerns (CS); one coder analyzed questions related to health and lifestyle (XW); and one coder analyzed questions related to coping mechanisms and barriers to treatment (AS). Between each phase of coding, the coders and other authors (SH and FS) met and discussed their process to ensure a uniform analysis method. The final coding structure and themes were decided upon in consensus meetings among all authors. Qualitative analyses were performed using Microsoft Excel (Microsoft Corp) and MAXQDA (VERBI Software) [15].

## Results

### Sample Demographics

A total of 2031 responses were collected, including 1252 (61.64%) from female respondents. Age of the participants ranged from 18 to 75 years (mean 22.88, SD 5.52). The sample included both undergraduate (n=1405, 69.18%) and graduate students (n=620, 30.53%), further classified as freshman (n=265, 13.05%), sophomore (n=274, 13.49%), junior (n=354, 17.43%), senior (n=512, 25.21%), master’s (n=294, 14.48%), and doctorate (n=326, 16.05%). Study program was reported by 1900 participants representing all 15 colleges in the Texas A&M campus. The top represented colleges were Engineering (n=565, 29.74%), Liberal Arts (n=261, 13.74%), and Agriculture and Life Sciences (n=189, 9.95%). Table 1 shows the gender, classification, and program (college) proportions of the sample compared to the Texas A&M population.

**Table 1:** Demographics.

Characteristic		Sample, n (%)		Population^a^, n (%)	
**Gender**					
	Female		1252 (61.64)		28,956 (46.60)
	Male		757 (37.27)		33,197 (53.40)
**Classification**					
	Undergraduate		1405 (69.18)		50,454 (81.18)
	Master’s		294 (14.48)		6259 (10.07)
	Doctorate		326 (16.05)		4864 (7.83)
**Age (years)**					
	<18		0 (0)		327 (0.53)
	18-21		1065 (52.44)		31,839 (51.23)
	22-25		532 (26.19)		22,946 (36.92)
	26-30		207 (10.19)		3947 (6.35)
	31-39		96 (4.73)		2176 (3.50)
	>39		46 (2.26)		918 (1.48)
**College**					
	College of Engineering		565 (29.74)		18,784 (30.22)
	College of Liberal Arts		261 (13.74)		8526 (13.72)
	College of Agriculture and Life Sciences		189 (9.95)		7473 (12.02)
	College of Education & Human Development		176 (9.26)		6630 (10.67)
	Mays Business School		154 (8.11)		6041 (9.72)
	College of Science		134 (7.05)		3828 (6.16)
	College of Veterinary Medicine & Biomedical Sciences		93 (4.89)		3575 (5.75)
	College of Architecture		78 (4.11)		3142 (5.06)
	School of Public Health		50 (2.63)		292 (0.47)
	College of Geosciences		49 (2.58)		1321 (2.13)
	Bush School of Government & Public Service		36 (1.89)		516 (0.83)

^a^Population based on Fall 2019 student demographics data at Texas A&M, College Station Campus.

### Severity of Depression and Anxiety

#### Depression

**Figure 1:**
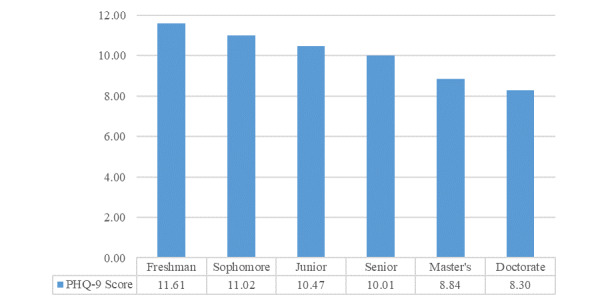
Mean Patient Health Questionnaire-9 (PHQ-9) score by classification.

A total of 37 responses were excluded from the PHQ-9 questionnaire analysis because of missing values. Among the 1994 complete responses, 1607 (80.57%) participants reported some (any) level of depression, as follows: mild (n=647, 32.45%), moderate (n=496, 24.87%), moderately severe (n=316, 15.85%), and severe (n=148, 7.42%). The two-way ANOVA showed that gender (*P*<.001, η^2^=0.03) and classification (*P*<.001, η^2^=0.03) significantly impacted PHQ-9 scores. Females had a mean score of 1.76 points higher than males (mean 10.61 and 8.84, respectively). Participants with a higher classification had lower PHQ-9 scores (Figure 1). Tukey’s honest significant difference (HSD) showed a significant difference between doctoral and all undergraduate classifications (*P*<.001, *P*<.001, *P*<.001, and *P*=.001, respectively), between master’s and freshman/sophomore/junior (*P*<.001, *P*<.001, and *P*=.04, respectively), and between senior and freshman (*P*=.004).

Responses to item 9 of the PHQ-9 (“over the last two weeks, how often have you been bothered by thoughts that you would be better off dead or of hurting yourself in some way?”) showed that 366 (18.04%) participants had thoughts related to self-harm or suicide (250 responded “several days,” 74 “more than half the days,” and 42 “nearly every day”).

#### Anxiety

**Figure 2:**
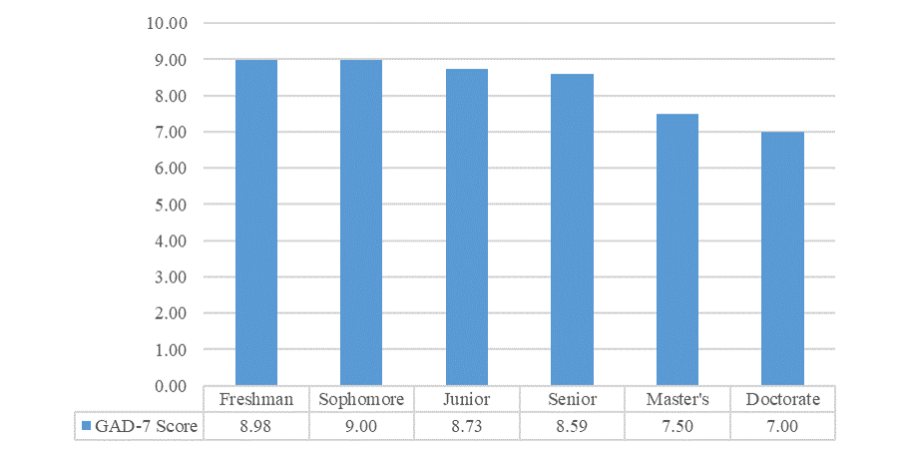
Mean Generalized Anxiety Disorder-7 (GAD-7) score by classification.

In total, 17 responses were excluded from the GAD-7 questionnaire analysis because of missing values. Among the 2014 complete responses, 569 (28.25%) participants reported minimal anxiety, while 71.75% (n=1445) showed anxiety, with severity levels varying as mild (n=670, 33.27%), moderate (n=477, 23.68%), or severe (n=298, 14.80%). The two-way ANOVA showed a significant main effect of gender (*P*<.001, η^2^=0.05) and classification (*P*<.001, η^2^=0.02) on GAD-7 score. Females had a mean score of 2.22 points higher than males (means scores were 9.12 and 6.89, respectively). Participants with a higher classification had lower GAD-7 scores (Figure 2). Tukey’s HSD test was conducted to test for differences between the classifications, and showed a significant difference between doctoral and all undergraduate classifications (*P*<.001, *P*<.001, *P*=.002, and *P*=.002, respectively), and between master’s and sophomore (*P*=.03).

### COVID-19–Related Stress

A majority of participants (n=1443, 71.26%) reported that their stress/anxiety levels had increased during the pandemic, while 111 (5.48%) indicated it had decreased and 471 (23.26%) indicated it remained the same as before. A vast majority (n=1982, 97.83%) of students thought other students were experiencing stress/anxiety because of the pandemic. Participants who indicated a change in their stress/anxiety levels were asked to specify reasons for such an increase or decrease in a follow-up question. Apart from the general comments on the stressors, participants also rated severity of the effect by specific academic-, health-, and lifestyle-related concerns.

#### Reasons for Increase in Stress

Among the participants who indicated increased stress/anxiety (n=1443) during the pandemic, 1360 participants elaborated on the reasons for such an increase. The biggest contributor was stress related to academics (532/1360, 39.12%), with the majority stemming from increased difficulty (n=278) due to the precipitous transition and maintenance of online classes, distantly followed by increased concerns over grades (n=58), and delayed graduation (n=53). The second most frequent contributor was general uncertainty regarding the pandemic (473/1360, 34.78%). This was followed by health concerns (472/1360, 34.71%) relating to personal mental health (n=205), health of friends and family (n=162), and fear of personally contracting COVID-19 (n=83). The fourth biggest concern related to finances (279/1360, 20.51%), primarily stemming from unemployment or uncertainty of future employment (n=183). Living/work environment (276/1360, 20.29%) was the next biggest contributor, consisting of concerns related to working from home (n=79), cabin fever (n=59), returning home (n=49), and confinement with others (n=47). Impacted social life (252/1360, 18.53%) was the last major category, primarily consisting of concerns related to isolation (n=186).

#### Reasons for Decrease in Stress

A few respondents (n=109) elaborated on the reasons that they were experiencing decreased stress and anxiety during the pandemic. The majority of these respondents mentioned that this was due to time saved (47/109, 43.1%) as a result of not having to commute to school, reduced schoolwork, and not having to engage in extracurricular and organizational activities, which are otherwise a part of campus life. A key benefit of the transition to distance learning was the schedule flexibility (n=20), particularly when lecture recordings could be viewed on students’ own time. Several students (n=13) also mentioned using the additional time available to pursue hobbies and other interests, as well as to amplify proactive health behaviors such as meditation and exercise. Interestingly, some students (n=8) reported that they were experiencing reduced social anxiety from not having to interact with other students.

#### Academic-Related Concerns

**Figure 3:**
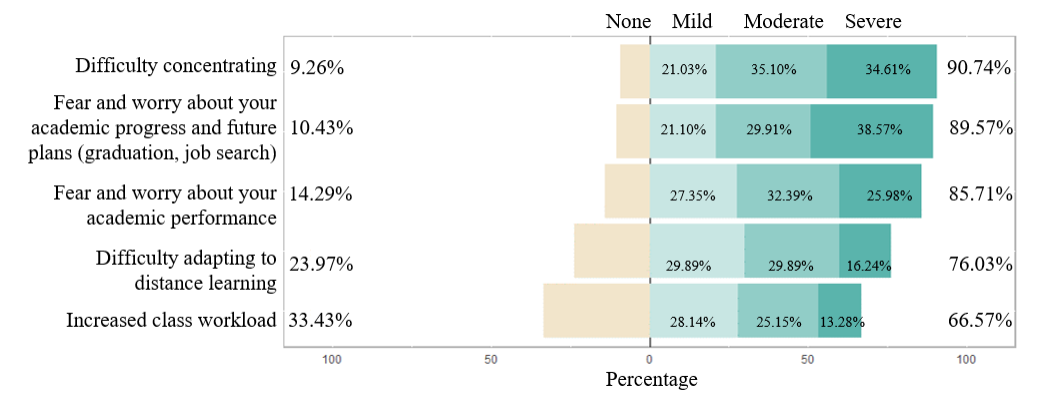
Academic-related concerns.

In terms of academic-related concerns (Figure 3), 1851 (90.74%) participants had difficulty in concentrating, with 716 (35.10%) rating their difficulty as moderate and 706 (34.61%) as severe. Similarly, most participants had concerns regarding their academic progress and future plans (n=1830, 89.57%), as well as academic performance (n=1752, 85.71%). A majority of the participants also had difficulty adapting to distance learning (n=1554, 76.03%) or had an increased class workload (n=1358, 66.57%).

A small portion of participants (n=389) provided additional free-response reasons behind the increased level of stress related to academics. Nearly one fifth of those who indicated such reasons (73/389, 18.8%) reported financial concerns related to their academic situations such as reduced current and prospective job opportunities (n=42), increased burden to pay tuition and fees (n=16), and impacts on scholarship and funding (n=15). Some of the participants (27/389, 6.9%) presented their worry about future semesters, such as continuing online classes in following semesters (n=12), choosing a major in the middle of the pandemic situation (n=10), and resuming in-person classes with persistent risks of virus infection (n=5).

#### Health-Related Concerns

**Figure 4:**
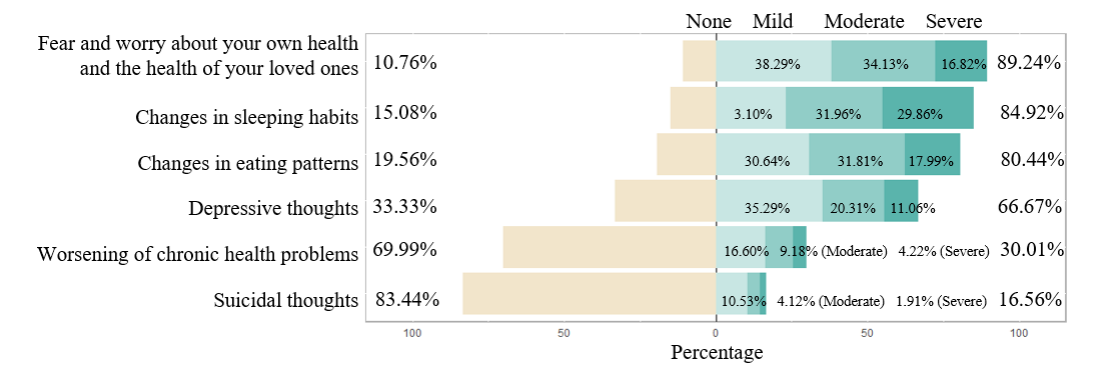
Health-related concerns.

As Figure 4 shows, the top health-related concern was fear and worry about personal health and the health of loved ones (n=1825, 89.24%), followed by changes in sleeping habits (n=1735, 84.92%), eating patterns (n=1641, 80.44%), and depressive thoughts (n=1362, 66.67%).

Some participants (n=180) added health-related concerns in the free responses. The biggest concern was physical illness (52/180, 29.9%), including having physical illness (n=31) that worsened during the pandemic (n=6). Some were at higher risk of getting infected with COVID-19 due to asthma (n=9), autoimmune disease (n=4), being immunocompromised (n=3) or other disease (n=3). Four participants reported that they have been infected with COVID-19. Fitness was another concern (49/180, 28.2%), including decreased exercise (n=45), weight gain (n=12), and muscle/back pain due to sedentary lifestyle (n=5). Some participants reported having been diagnosed with mental illness (n=31), which worsened during the pandemic (n=11). Barriers to health care were mentioned by 29 participants, including barriers or reluctance to visit doctors for nonpandemic issues (n=24), barriers to get medication (n=3), and barriers to get tested for COVID-19 (n=2).

#### Lifestyle-Related Concerns

**Figure 5:**
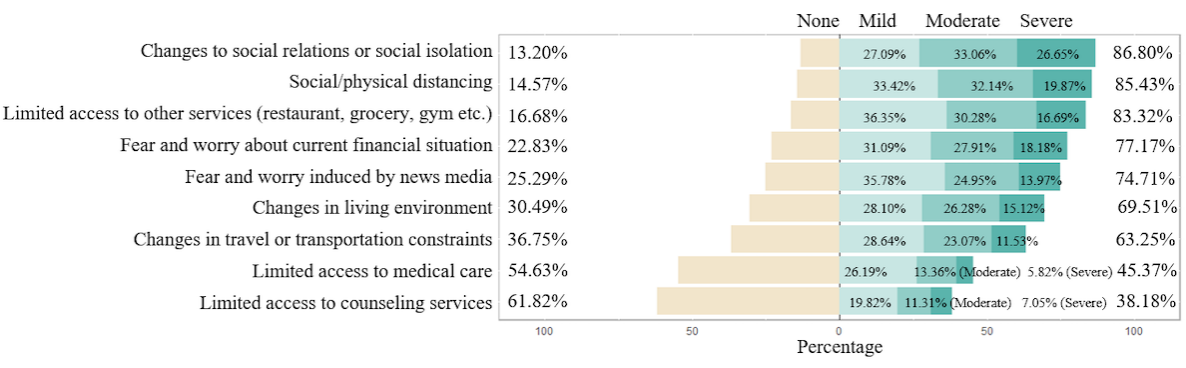
Lifestyle-related concerns.

More than half of the participants reported experiencing the top seven lifestyle concerns shown in Figure 5. It was not surprising that “changes to social relations or social isolation” (n=1775, 86.80%) and “social/physical distancing” (n=1741, 85.43%) were the top two lifestyle concerns.

Some participants (n=174) mentioned additional concerns related to lifestyle changes in the free responses. The major concern was relationship (71/174, 40.8%), including social activities affected by isolation (n=45) and relationship issues with family members (n=18) and roommates (n=8). Some participants had difficulty working (n=17), due to unsatisfying work environments (n=9) such as internet issues (n=2), or had difficulty following a schedule (n=8). Other concerns included uncertainty of the future (n=12), worry because others do not perform social distancing (n=11), and worry of bringing the virus to their family as essential workers (n=2).

### Coping Mechanisms and Barriers

#### Coping Mechanisms

Nearly half (n=882, 43.25%) of the participants indicated via multiple-choice responses that they were able to cope adequately with the stress related to the current situation, while 323 (15.84%) said they were not able to cope. The rest were unsure (n=834, 40.90%). When asked what coping mechanisms were used to mitigate stress/anxiety, more than half (n=1362, 67.06%) of the participants chose “support from community, family and friends,” followed by “technologies (websites, mobile apps, sensors that help monitor health data)” (n=659, 32.45%). Few participants reported using university health services such as counseling service (n=210, 10.34%) or health services outside the university (n=89, 4.38%). Some of the participants (n=387, 19.05%) reported using no coping mechanism.

Some respondents (n=386) indicated that they used other coping mechanisms to reduce stress during the COVID-19 and provided elaboration in the free-response field. Many of these respondents (152/386, 39.0%) mentioned engaging in health lifestyle activities such as exercise (n=130), diet maintenance (n=11), and self-care activities (n=11). A similar number of respondents (143/386, 37.1%) engaged in relaxing activities including meditation (n=48), reading (n=21), playing with pets (n=13), listening to music (n=12), breathing exercises (n=4), sleeping (n=3), shooting for sport (n=2), gardening (n=1), and other hobbies in general (n=29) alongside general relaxing activities (n=10). Beyond relaxing activities, some respondents engaged in creative activities (31/386, 8.0%), which included creating pieces of art (n=10), writing (n=18), and playing musical instruments (n=3). Additionally, respondents mentioned engaging in spiritual and religious activities (n=69) such as reading sacred texts and praying. A small percentage of the respondents also engaged in negative coping methods (41/386, 10.6%), including distracting themselves (n=20), excessive intake of alcohol (n=5), isolation (n=2), auto-manipulation (n=2), and crying (n=1). Lastly, some respondents maintained focus on their work and professional activities (16/386, 4.2%) by concentrating on school (n=4), continuing work (n=3), or managing their time for productive use.

#### Smartphone Apps for Coping With COVID-19

A small number of participants (n=290, 14.28%) indicated that they have been using mobile apps for managing added stress related to the pandemic, 278 of whom provided specific names of the apps or features. Most of these respondents (201/278, 72.3%) used an app focused on mindfulness. Most of these mindfulness apps focused on meditation (144), such as Headspace; relaxation apps (n=42) and apps centered on focused breathing (n=15) were also used. Social media (46/278, 16.5%) was used by some of the respondents with most using the typical social media sites (eg, Twitter, Facebook, Instagram, and TikTok) and apps (n=32) for entertainment purposes while others used YouTube (n=14). Some respondents used lifestyle apps (37/278, 13.3%), including exercise apps (n=23), time management (n=9), sleep tracking (n=5), and food tracking (n=2). A small number of respondents (6/278, 2.2%) played video game apps to cope with stress during COVID-19.

#### Sources of Information for COVID-19

The main sources of information related to the pandemic were university emails (n=1257, 61.89%), newspapers, paper or online periodicals (n=1221, 60.12%), and posts on social media (n=1026, 50.52%). Some participants also received information from medical websites (n=591, 29.10%) or their medical providers (n=315, 15.51%).

A small number of respondents (n=235) elaborated on the other information sources in open-ended responses. About a quarter (60/235, 25.5%) of these respondents received their information from more traditional media including television (n=34), online articles (n=19), and the radio (n=7). A similar number of participants (55/235, 23.4%) received their information from family and friends. Other respondents listened to figures of authority (50/235, 21.3%) for their information regarding COVID-19, split between government officials and organizations, including presidential briefings and local leaders (n=32) on one hand, and prominent scientists and researchers (n=28) on the other. Finally, some respondents preferred to rely on themselves to stay informed (21/235, 8.9%) by conducting searches and reading relevant sources (n=4), or by ignoring the news due to disbelief in or indifference toward the current situation (n=17).

#### Barriers for Mental Health Care

The major barriers to mental health care were “financial concerns (fees and insurance)” (n=1434, 70.61%), “social stigma” (n=1194, 58.79%), “lack of information about resources available” (n=1099, 54.11%), and “limited access to the services (eg, could not get scheduled)” (n=978, 48.15%).

A few respondents (n=167) indicated that they experienced other barriers and elaborated through the free-response section. Some of the respondents perceived that they themselves (69/167, 41.3%) can be the biggest barrier to reaching out for help: some doubted the efficacy of care (n=8); some mentioned that they or others may not see a problem even if it exists (n=22); others mentioned not wanting help (n=7). Respondents cited an overall feeling of discomfort (25/167, 15.0%) with the topic of mental health and difficulty bringing up the topic with others. Some of the respondents mentioned poor quality (22/167, 13.2%) in their treatment as a barrier to seeking treatment again.

## Discussion

### Principal Findings

Among the 2031 participants, 48.14% showed a moderate-to-severe level of depression, 38.48% showed a mild-to-severe level of anxiety, and 18.04% had suicidal thoughts in the 2 weeks preceding the survey. Gender and classification had significant effects on depression and anxiety severity (*P*<.001). Female respondents reported higher scores, while respondents in a higher classification reported lower scores on PHQ-9 and GAD-7. A majority of participants (71.26%) indicated that their stress/anxiety levels had increased during the pandemic. Less than half (43.25%) indicated that they were able to cope adequately with the stress related to the current situation.

The survey had a healthy representation across genders and classifications of undergraduate and graduate students. A vast majority (80.57%) of respondents had scores on the PHQ-9 that indicated some level of depression (defined as a total PHQ-9 score of ≥5), with about 48% in the moderate-to-severe range. This proportion of respondents showing depression is much larger than those found in recent assessments in China. For instance, in their survey of 509 college students, Liu et al [16] found that about 19% of their respondents showed some level of depression. Our findings also show a higher proportion of respondents with depressive symptoms among students than findings in several recent studies in nonpandemic situations [17,18]. Furthermore, nearly 1 in 5 respondents reported having suicidal thoughts. This finding is in line with the increased suicide rates observed during previous pandemics [19]. In comparison, previous research has reported about 3% to 7% of the college student population had suicidal thoughts outside of a pandemic situation [20]. This is an alarming finding warranting immediate attention. Additionally, a majority of our respondents (71.75%) showed some level of anxiety (defined as a total GAD-7 score of ≥5), with over 38% in the moderate-to-severe range. Again, this is a much higher proportion compared to similar survey-based assessments by Liu et al [16] and Cao et al [9], who found some level of anxiety in 8.8% (out of 509) and 24.9% (out of 7143) of respondents, respectively. Clearly, there is a pressing need to actively provide support to vulnerable students in managing their mental health.

Not surprisingly, given the above findings, a majority of respondents reported that their stress and anxiety had increased during the pandemic. In general, this is consistent with the heightened levels of psychological distress reported among various populations during the current pandemic and previous epidemics such as severe acute respiratory syndrome (SARS) [6,7,9,21]. This finding could be underpinned by the high levels of academic, health, and lifestyle concerns and changes. A vast majority indicated difficulty concentrating, fear, and worry about academic progress and performance, and adjustment to distance learning as dominant academic concerns. To our knowledge, this is the first study that reports these specific effects of the COVID-19 pandemic as related to academic concerns. Given that several universities, including Texas A&M, are continuing partially with distance learning for the remainder of the year, these concerns need to be probed further in order to be adequately addressed.

Among health-related concerns, a majority of students expressed concerns about their own health or the health of loved ones, echoing recent findings [6]. A large proportion (over 80%) of respondents reported changes in eating and sleeping habits. Again, this is not surprising, but certainly concerning, given previous research which has shown that such changes are correlated with depression among college students [22]. Among lifestyle-related concerns, physical distancing and changes in social relations were widely reported, similar to those found earlier among students as well as the general population [6,23]. Additionally, three quarters of respondents indicated fear and worry induced by news outlets. This type of distress may be exacerbated by the large amount of misinformation, including false and fabricated information, distributed through news and social media platforms [24].

More than half the respondents who described coping mechanisms mentioned support from family and friends as a key factor, similar to previous findings [6,9]. Several respondents also mentioned the use of technology, such as mobile apps and other digital platforms, as a means of positive coping practices, such as meditation, echoing recent findings on the positive effect of a mindfulness app on college students’ mental health [25]. This indicates some potential for mobile-based technologies to support mental health. Such platforms may have the added benefit of helping overcome the barrier of social stigma related to seeking help from counseling services. Identifying such positive coping behaviors is important in order to enable those behaviors through symptoms-level support.

### Limitations and Future Work

Several limitations may impact the generalizability of the findings reported in this paper. Most importantly, some of our findings may be biased due to self-selection by respondents. The higher percentage of respondents with depression/anxiety may be related to this bias. Particularly, the slightly higher level of depression/anxiety among females may be attributed to the slightly higher percentage of female respondents. Additionally, we did not ask if respondents had any existing mental health issues or were receiving treatment before the pandemic. In fact, 31 respondents mentioned on their own that they had a prior diagnosis of mental illness. Therefore, we are unable to clarify whether our findings have been biased by a population of respondents with pre-existing or heightened levels of distress, who may have been more inclined to participate in the survey than those who were less distressed.

The survey is cross-sectional and lacks comparison to a typical semester unaffected by the pandemic, or a different time point of the year. Huckins et al [26] tracked depression and anxiety severity of 217 undergraduate students using PHQ-4 and GAD-2 during Spring 2020. It was found that depression and anxiety levels spiked when the campus switched to remote learning but decreased in the following 2 weeks. It is valuable to keep monitoring the change to understand the long-term effect of the pandemic.

An interesting finding is the differences in depression/anxiety levels among different classifications of students. Undergraduate students may have been more heavily impacted during the pandemic compared to graduate students, probably from adapting to distance learning. Yet the precise factors need further investigation in a future study.

The proportion of respondents showing depression, anxiety, and/or suicidal thoughts is alarming. Respondents reported academic, health, and lifestyle-related concerns caused by the pandemic. Given the unexpected length and severity of the pandemic, these concerns need to be further understood and addressed. Further study on the most at-risk populations and evidence-based interventions should proceed as soon as possible to prevent a secondary epidemic, embedded within the COVID-19 pandemic, of a serious, nationwide mental affliction and potential physical self-harm among vulnerable college students.

At an institutional level, online remote activities and services can be implemented to provide support to students that help address concerns related to the pandemic. For example, Schlesselman et al [27] provided a list of activities that can potentially support students in fitness, socialization, and academic success (eg, virtual group exercise, virtual movie night, and virtual office hours). However, there is no one-size-fits-all solution. More work is needed to identify appropriate ways to implement such support and assess the long-term effects of such interventions.

## Abbreviations

ANOVA: analysis of variance
GAD-7: General Anxiety Disorder 7-item
PHQ-9: Patient Health Questionnaire 9-item
SARS: severe acute respiratory syndrome
Tukey’s HSD: Tukey’s honest significant difference
